# Dose reporting and dose-stratified effects of early mobilization in mechanically ventilated adult ICU patients: a systematic review and meta-analysis

**DOI:** 10.3389/fmed.2026.1920374

**Published:** 2026-08-31

**Authors:** Hong Zhou, Shengting Li, Tingting Tu, Xiulan Yang, Mingtao Quan, Zhihui Shang, Xia Zhang

**Affiliations:** 1 Department of Critical Care Medicine, Affiliated Hospital of Zunyi Medical University, Zunyi, China; 2 School of Nursing, Zunyi Medical University, Zunyi, China; 3 School of Medical Information Engineering, Zunyi Medical University, Zunyi, China

**Keywords:** dose-response relationship, early ambulation, intensive care units, meta-analysis, respiration, artificial

## Abstract

**Background:**

This systematic review and meta-analysis aimed to evaluate how early mobilization dose is reported and to explore dose-stratified effects of early mobilization on functional recovery, clinical outcomes, diaphragmatic function, and safety in mechanically ventilated adult ICU patients.

**Methods:**

Eight electronic databases were searched from inception to May 2026. Randomized controlled trials involving mechanically ventilated adult intensive care unit patients were included. Two reviewers independently screened studies, extracted data, and assessed risk of bias using the Cochrane Risk of Bias tool. Early mobilization interventions were classified as low-, moderate-, or high-dose according to frequency, duration, and intensity.

**Results:**

Thirty-one randomized controlled trials involving 3,498 participants were included. Overall, early mobilization improved functional and selected clinical outcomes, without significantly affecting mortality [RR = 1.13, 95% CI (0.89, 1.42), *P* = 0.258]. In dose-stratified analyses, the moderate-dose subgroup showed the most favorable effects on ICU-acquired weakness [RR = 0.19, 95% CI (0.09, 0.41)] and ICU length of stay [MD = −4.14, 95% CI (−5.00, −3.27), *P* < 0.001]. For adverse events, significant reductions were observed in the moderate-dose [RR = 0.41, 95% CI (0.25, 0.67), *P* < 0.001] and low-dose subgroups [RR = 0.25, 95% CI (0.11, 0.57), *P* = 0.001], but not in the high-dose subgroup [RR = 0.85, 95% CI (0.21, 3.54), *P* = 0.822].

**Conclusions:**

Incomplete and inconsistent reporting of mobilization dose may partly explain the variability in meta-analytic findings across early mobilization trials. Moderate- to high-dose mobilization appears to be associated with more favorable outcomes when tailored to clinical resources and patient tolerance. Given the substantial heterogeneity across studies, future trials should standardize the definition and reporting of mobilization dose to support individualized and resource-optimized mobilization strategies.

**Systematic Review Registration:**

PROSPERO, CRD420261416276.

## 1 Introduction

Approximately 30%–90% of patients admitted to intensive care units (ICUs) worldwide require mechanical ventilation (MV) (1). Although mechanical ventilation is a life-sustaining intervention, prolonged immobilization during ventilatory support restricts out-of-bed activity and increases the risk of ICU-related physical, psychological, and cognitive impairments (2–4). Early mobilization has been suggested as a promising intervention to counteract ICU-AW and mitigate the adverse consequences of prolonged bed rest (5–7). It has increasingly been incorporated into ICU care, with mobilization protocols commonly including progressive mobility, passive or active exercises, in-bed or bedside cycling, neuromuscular electrical stimulation, functional electrical stimulation, and out-of-bed activities (8).

However, despite growing evidence supporting early mobilization, substantial heterogeneity remains across intervention protocols (9). Important components of mobilization dose, including timing, frequency, duration, intensity, progression criteria, and coordination with other ICU treatments, are inconsistently defined and reported (10). In many studies, only the highest level of mobilization achieved is quantified, whereas the duration, frequency, and intensity of mobilization are not systematically captured (11). This limits the ability to determine whether different mobilization programs produce comparable therapeutic effects (12).

Moreover, mechanically ventilated ICU patients represent a highly heterogeneous population (13). Factors such as illness severity, age, baseline functional status, frailty, comorbidities, sedation depth, respiratory support requirements, and treatment invasiveness may influence both tolerance to mobilization and the response to rehabilitation (14). Consequently, it remains difficult to determine the optimal timing and dose of early mobilization that maximize benefit while maintaining safety for individual patients (15).

An individualized and dose-informed approach to early mobilization may therefore be essential for optimizing rehabilitation in mechanically ventilated ICU patients (16). However, previous reviews have mainly synthesized the overall effects of early mobilization, whereas the way in which mobilization dose is reported and whether different reported dose levels are associated with different outcomes remain insufficiently clarified (17–19). Accordingly, this systematic review and meta-analysis aimed to synthesize mobilization dose reporting and to explore dose-stratified effects of early mobilization in mechanically ventilated adult ICU patients on functional recovery, clinical outcomes, diaphragmatic function, and safety.

## 2 Methods

This systematic review was reported in accordance with the Preferred Reporting Items for Systematic Reviews and Meta-Analyses guidelines (20). Additionally, the protocol was prospectively registered in the International Prospective Register of Systematic Reviews (PROSPERO; registration number: CRD420261416276) to ensure transparency and reduce the risk of reporting bias. Ethics approval was not required because this systematic review and meta-analysis was based exclusively on previously published studies and did not involve individual patient data, human biological samples, or identifiable personal information.

### 2.1 Inclusion and exclusion criteria

The research question was structured using the PICOS framework. Regarding the population (P), adult ICU patients receiving mechanical ventilation (MV) at enrolment or during the ICU stay were considered. The intervention (I) involved the use of early mobilization during the ICU stay, compared (C) with conventional treatment. The outcomes (O) associated with this intervention were assessed, and the study design (S) was restricted to randomized controlled trials (RCTs).

Inclusion criteria: (i) the target population was critically ill adult patients receiving MV in the ICU at enrollment or during the intervention window; (ii) study design: RCTs; (iii) intervention: early mobilization and rehabilitation initiated during the ICU stay, with sufficient information on mobilization frequency, duration, intensity, or activity level to allow dose classification; and (iv) the outcomes analysed included muscle strength, diaphragmatic function, duration of mechanical ventilation, length of stay in the ICU and in the hospital, functional status, mortality, and adverse events.

Exclusion criteria: (i) the population consisted of individuals under the age of 18 years; (ii) studies were published in languages other than English or Chinese; (iii) exercises were performed after ICU discharge; (iv) reviews, abstracts, and case reports; and (v) animal or cell-based studies and duplicate publications.

### 2.2 Search strategy

We searched electronic databases, including PubMed, Web of Science, Cochrane Library, Embase, China National Knowledge Infrastructure (CNKI), Chinese Biomedical Literature Service System (SinoMed), China Science and Technology Journal Database (VIP), and Wanfang Database, from database inception to May 2026. We used a combination of controlled vocabulary (MeSH terms for PubMed) and keywords to conduct our search and also searched reference lists from included studies for additional relevant articles. The full search strategies for all databases are provided in Supplementary Table S1.

### 2.3 Literature screening and data extraction

EndNote software was used to manage the retrieved citations. Two researchers independently screened titles and abstracts, assessed full texts for eligibility, extracted data, and cross-checked their results using a predesigned and pilot-tested data extraction form. Any disagreements were resolved through discussion with a third reviewer. The extracted information included basic study characteristics, such as first author, year of publication, and country; participant characteristics, such as medical conditions, sedation control, mean age, and sample size; intervention characteristics of the experimental group, such as program name, program components, intervention timing, and delivery frequency; intervention characteristics of the control group; and early mobilization-related outcomes.

### 2.4 Data management

The types of treatment were grouped into five categories according to the intervention program: (i) Conventional physical therapy (CPT), which included positioning, stretching, range of motion exercise, and chest physical therapy, such as manual hyperinflation, chest expansion exercise, postural drainage, and suction. (ii) Exercise-based physical therapy (EPT), which included strengthening, balance training, and cycling. (iii) Neuromuscular electrical stimulation (NMES). (iv) Progressive mobility (PM), in which the program was progressed according to patient ability, from passive range of motion for patients unable to follow instructions to sitting, sitting on the edge of the bed, transferring to a chair, standing, stepping in place, and walking. (v) protocolized or multicomponent early mobilization (PMEM), in which multiple treatment elements were integrated into a structured or bundled early mobilization program.

Because no universally accepted standard exists for defining the dose of early mobilization in mechanically ventilated ICU patients, the dose classification in this review was developed *a priori* as an operational framework based on previous studies on mobilization dose reporting (21, 22), the frequency, intensity, time, and type (FITT) principle of exercise prescription (23), and the available dose-related information reported in the included trials, including intervention frequency, daily duration, activity level, and NMES duration. These categories were used to support exploratory subgroup analyses.

For categorizing treatment dose, studies were classified according to the prescribed or reported average dose on scheduled intervention days during the intervention period. Studies were classified as follows: (i) high-dose early mobilization, defined as ≥ 2 sessions per day or NMES for ≥60 min per day; (ii) moderate-dose early mobilization, defined as one session per day for 3–7 days per week or NMES for 30–60 min per day; and (iii) low-dose early mobilization, defined as < 3 sessions per week, an activity duration of<20 min per day, or NMES for <30 min per day.

### 2.5 Risk-of-bias assessment

We assessed the risk of bias in RCTs using the Cochrane risk-of-bias tool (24). Each trial was regarded as having a high, unclear, or low risk of bias based on the following criteria: random sequence generation, allocation concealment, blinding of participants and personnel, blinding of outcome assessment, incomplete outcome data, selective reporting, and other bias.

### 2.6 Statistical analysis

Risk ratios (RRs) with 95% confidence intervals (CIs) were calculated for dichotomous outcomes, and mean differences (MDs) with 95% CIs were calculated for continuous outcomes. When outcomes were measured using different scales, standardized mean differences (SMDs) with 95% CIs were used. Heterogeneity was assessed using the *I*^2^ statistic and the Cochrane *Q*-test. A *P* value < 0.10 for the *Q*-test or an *I*^2^ value > 50% was considered to indicate significant heterogeneity. When substantial heterogeneity was identified, a random-effects model was used to summarize the data; otherwise, a fixed-effects model was applied. Dose-stratified subgroup analyses were performed according to the predefined dose categories, and between-subgroup differences were assessed using the *χ*^2^-test. When sufficient studies were available, meta-regression was conducted to explore whether mobilization dose contributed to heterogeneity. Sensitivity analysis was performed to examine the influence of individual studies on the pooled results. Publication bias was assessed using Begg's and Egger's tests when at least 10 studies were available for an outcome. A *P* value < 0.05 was considered statistically significant, except where otherwise specified. All analyses were performed using Stata version 18.0 (StataCorp LLC, College Station, TX, USA).

## 3 Results

**Figure 1:**
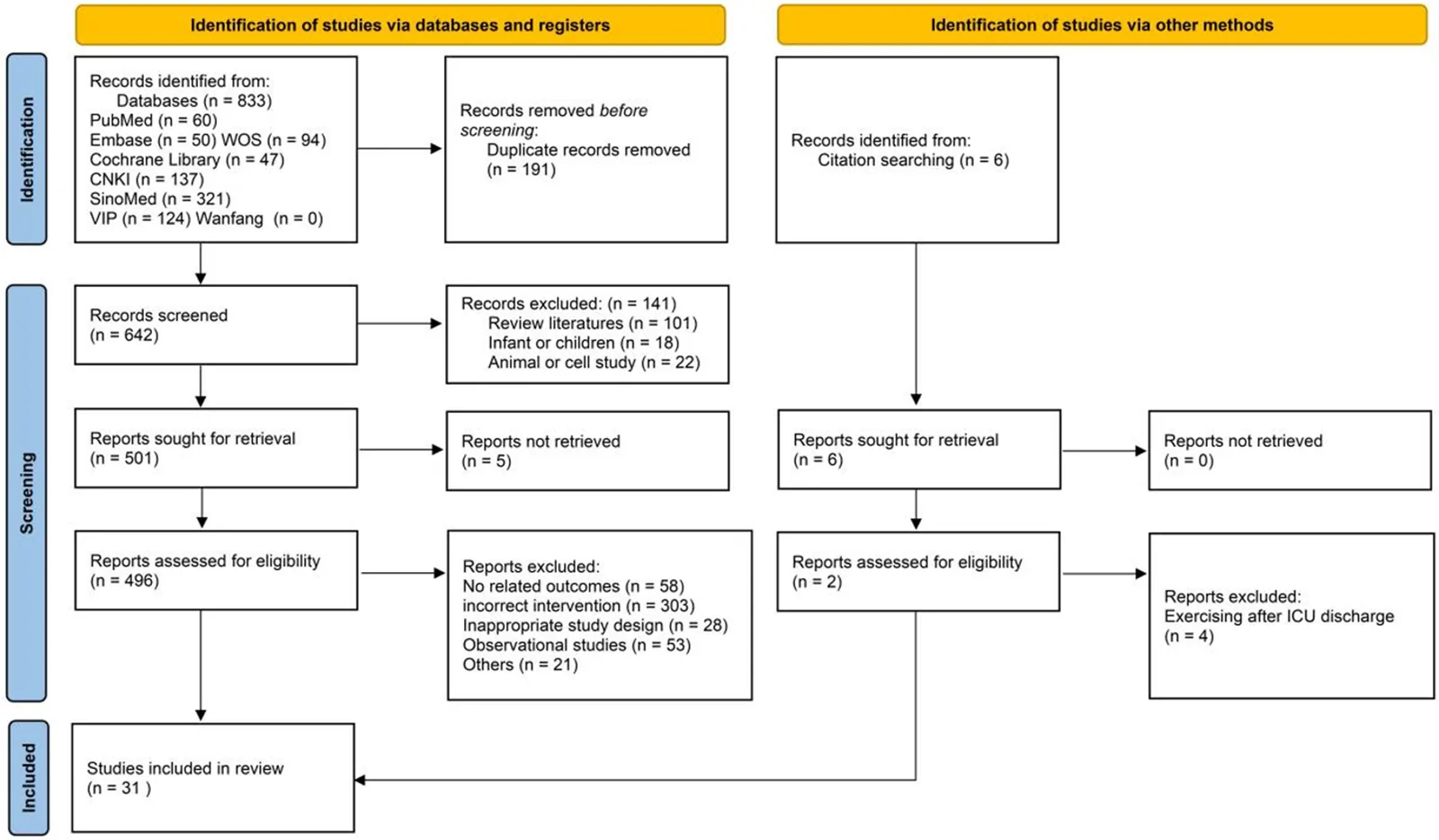
PRISMA 2020 flow diagram of study selection. PRISMA: Preferred Reporting Items for Systematic Reviews and Meta-Analyses.

Figure 1 presents the Preferred Reporting Items for Systematic Reviews and Meta-Analyses flow diagram, illustrating the stages of the study selection process. In total, 31 RCTs that met the eligibility criteria were included and underwent a detailed process of data extraction and methodological quality assessment.

### 3.1 Study characteristics

The main characteristics of the included studies are summarized in Table 1. A total of 31 randomized controlled trials (25–55) involving 3,498 mechanically ventilated adult ICU patients were included, with sample sizes ranging from 50 to 741 participants. Most studies were conducted in China (*n* = 24) (25–28, 30–32, 34–43, 45, 46, 48–50, 53, 54), while the remaining studies were from Egypt (29), Canada (44), Switzerland (47), the United States (55), or multiple countries (51, 52). Overall, 2,053 participants were male and 1,445 were female.

**Table 1:** Characteristics of the included studies.

Study	Country	Sample size (*N*)	Gender (M/F)	Intervention type	Participants	Dose group
Pi Xuezhen (25)	China	120	73/47	PMEM	ICU	High
Zhou Jie et al. (26)	China	104	53/51	PMEM	ICU	Moderate
Zhang Rui et al. (27)	China	80	55/25	PMEM	ICU	High
Ma Qingyun et al. (28)	China	80	49/31	PMEM	ICU	Low
Wu Hualian et al. (30)	China	138	71/67	PM	ICU	Low
Liu Xiu et al. (31)	China	64	40/24	PMEM	ICU	Low
Luo Wei et al. (34)	China	68	43/25	EPT	ICU	High
Chen Peiying et al. (32)	China	86	46/40	PMEM	ICU	Low
Liu Chunfeng (35)	China	138	76/62	PMEM	ICU	Low
Wang Lai et al. (36)	China	78	40/38	PM	ICU	High
Qin Zhonghui et al. (37)	China	84	57/27	PMEM	ICU	Moderate
Lu Hong (38)	China	88	47/41	EPT	ICU	Moderate
Bian Hong (39)	China	56	27/29	PMEM	ICU	Moderate
Zhang Hao et al. (40)	China	77	53/24	CPT	ICU	Moderate
Shao Xiaoji (41)	China	90	50/40	PMEM	ICU	Low
Wang Yuanfeng et al. (42)	China	112	66/46	PM	ICU	Moderate
Zhong Jingliang et al. (43)	China	104	55/49	PM	ICU	High
Dou Yingru et al. (45)	China	120	70/50	EPT	ICU	Moderate
Yang Shengqiang et al. (46)	China	60	34/26	CPT	ICU	Low
Feng Min et al. (48)	China	56	34/22	PM	RICU	Low
Wu Hualian et al. (49)	China	101	51/50	PMEM	ICU	Low
Huang Haiyan et al. (50)	China	100	53/47	PMEM	ICU	Moderate
Dong Zehua et al. (53)	China	60	41/19	CPT	ICU	Low
Hu Xiling et al. (54)	China	98	56/42	CPT	ICU	Moderate
Othman et al. (29)	Egypt	60	30/30	NMES	ICU	Moderate
TEAM Study Investigators (33)	Multiple countries	741	467/274	PMEM	ICU	High
Kho et al. (44)	Canada	66	31/35	PMEM	ICU	Moderate
Eggmann et al. (47)	Switzerland	115	77/38	EPT	ICU	High
Hodgson et al. (51)	Multiple countries	50	30/20	PMEM	ICU	Low
Schaller et al. (52)	Multiple countries	200	126/74	PMEM	SICU	High
Schweickert et al. (55)	United States	104	52/52	PMEM	ICU	Low

CPT, conventional physical therapy; EPT, exercise-based physical therapy; PM, progressive mobility; NMES, neuromuscular electrical stimulation; PMEM, protocolized or multicomponent early mobilization. ICU, intensive care unit; RICU, respiratory intensive care unit; SICU, surgical intensive care unit.

Most participants were recruited from general ICUs (25–47, 49–51, 53–55), with one study conducted in a respiratory ICU (48) and one in a surgical ICU (52). Protocolized or multicomponent early mobilization was the most common intervention type (*n* = 17) (25–28, 31–33, 35, 37, 39, 41, 44, 49–52, 55), followed by progressive mobility (*n* = 5) (30, 36, 42, 43, 48), exercise-based physical therapy (*n* = 4) (34, 38, 45, 47), conventional physical therapy (*n* = 4) (40, 46, 53, 54), and neuromuscular electrical stimulation (*n* = 1) (29). According to the predefined dose-stratification criteria, 8 studies were classified as high-dose early mobilization (25, 27, 33, 34, 36, 43, 47, 52), 11 as moderate-dose early mobilization (26, 29, 37–40, 42, 44, 45, 50, 54), and 12 as low-dose early mobilization (28, 30–32, 35, 41, 46, 48, 49, 51, 53, 55).

### 3.2 Risk of bias

**Figure 2:**
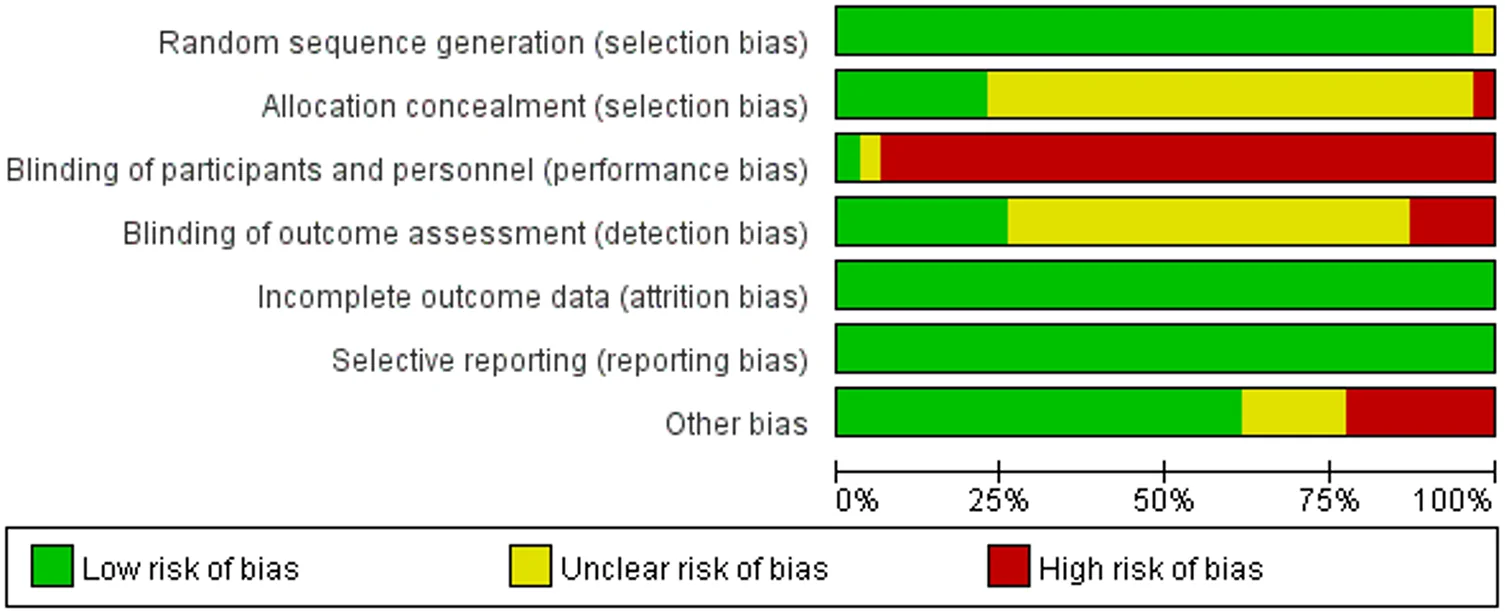
Risk of bias graph.

The overall risk-of-bias assessment is shown in Figure 2. Most studies adequately described random sequence generation, with 28 studies (90.3%) rated as low risk (25–32, 34–36, 38–54). Allocation concealment was unclear in 20 studies (64.5%) (26–31, 34–38, 40–43, 45, 46, 48–50, 53, 54). Because blinding of participants and personnel is inherently difficult in early mobilization interventions, 26 studies (83.9%) were judged to be at high risk of performance bias (25–29, 32, 34–46, 48–50, 52–55). Incomplete outcome data and selective reporting were rated as low risk in all included studies. Although several domains were rated as low or unclear risk, the frequent lack of blinding should be considered an important methodological limitation when interpreting the robustness of the pooled findings. The domain-level judgments for each included study are presented in Supplementary Figure S1.

### 3.3 Functional Status outcomes

**Figure 3:**
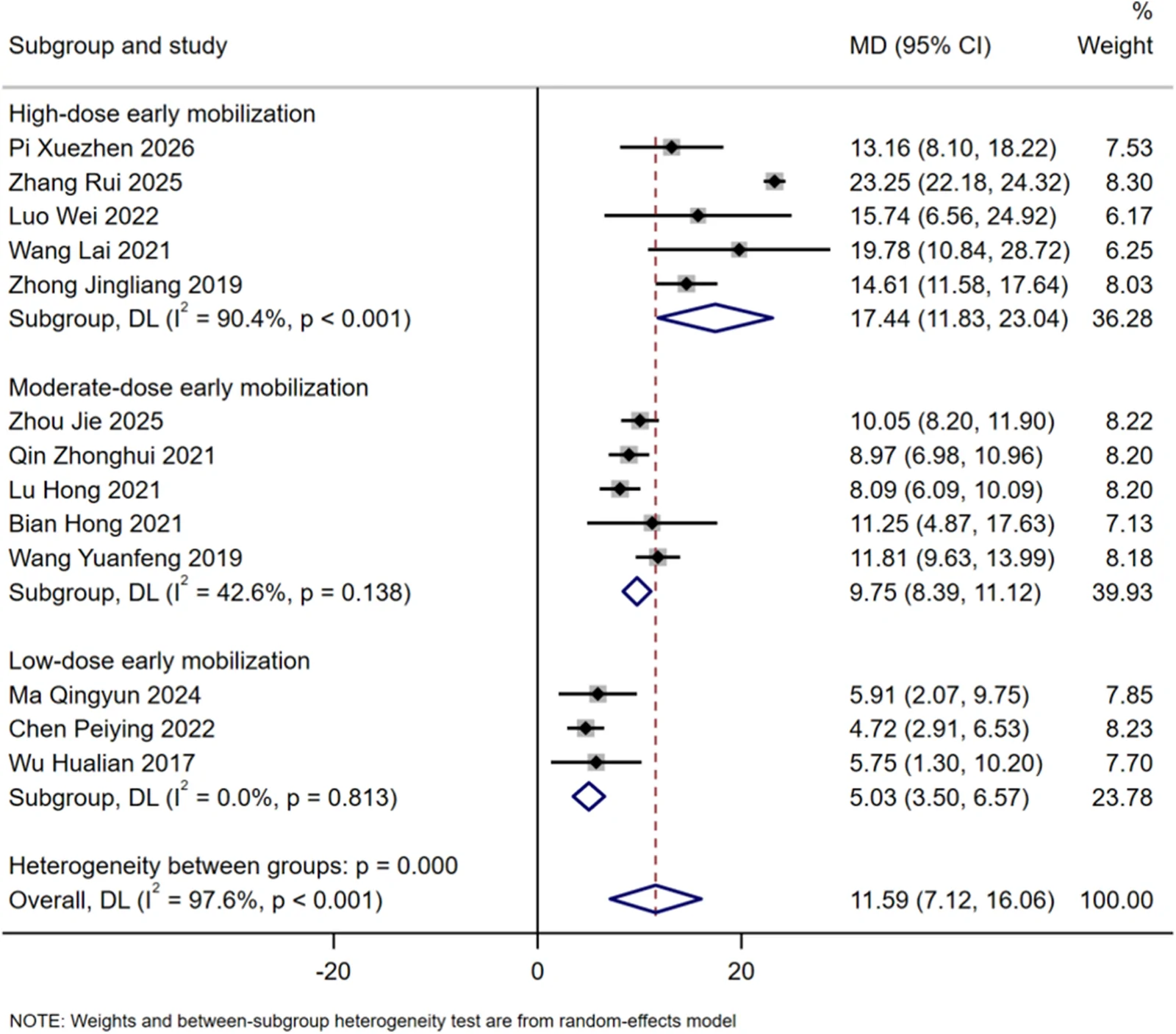
BI forest plot.

For activities of daily living assessed using the Barthel Index (BI), pooled analysis showed that early mobilization significantly improved BI scores compared with standard care [MD = 11.59, 95% CI (7.12, 16.06), *P* < 0.001; Figure 3]. Dose-stratified subgroup analysis revealed a graded effect. High-dose early mobilization yielded the greatest improvement [MD = 17.44, 95% CI (11.83, 23.04), *P* < 0.001], followed by moderate-dose [MD = 9.75, 95% CI (8.39, 11.12), *P* < 0.001] and low-dose interventions [MD = 5.03, 95% CI (3.50, 6.57), *P* < 0.001].

The heterogeneity test indicated substantial overall heterogeneity (*I*^2^ = 97.6%, *P* < 0.001). Within-subgroup heterogeneity was high in the high-dose subgroup (*I*^2^ = 90.4%, *P* < 0.001), moderate in the moderate-dose subgroup (*I*^2^ = 42.6%, *P* = 0.138), and absent in the low-dose subgroup (*I*^2^ = 0.0%, *P* = 0.813). The test for between-subgroup heterogeneity was statistically significant (Q = 31.50, df = 2, *P* < 0.001). Meta-regression further indicated that mobilization dose was a significant contributor to the observed heterogeneity (*P* < 0.001).

### 3.4 Muscle strength and ICU-acquired weakness

**Figure 4:**
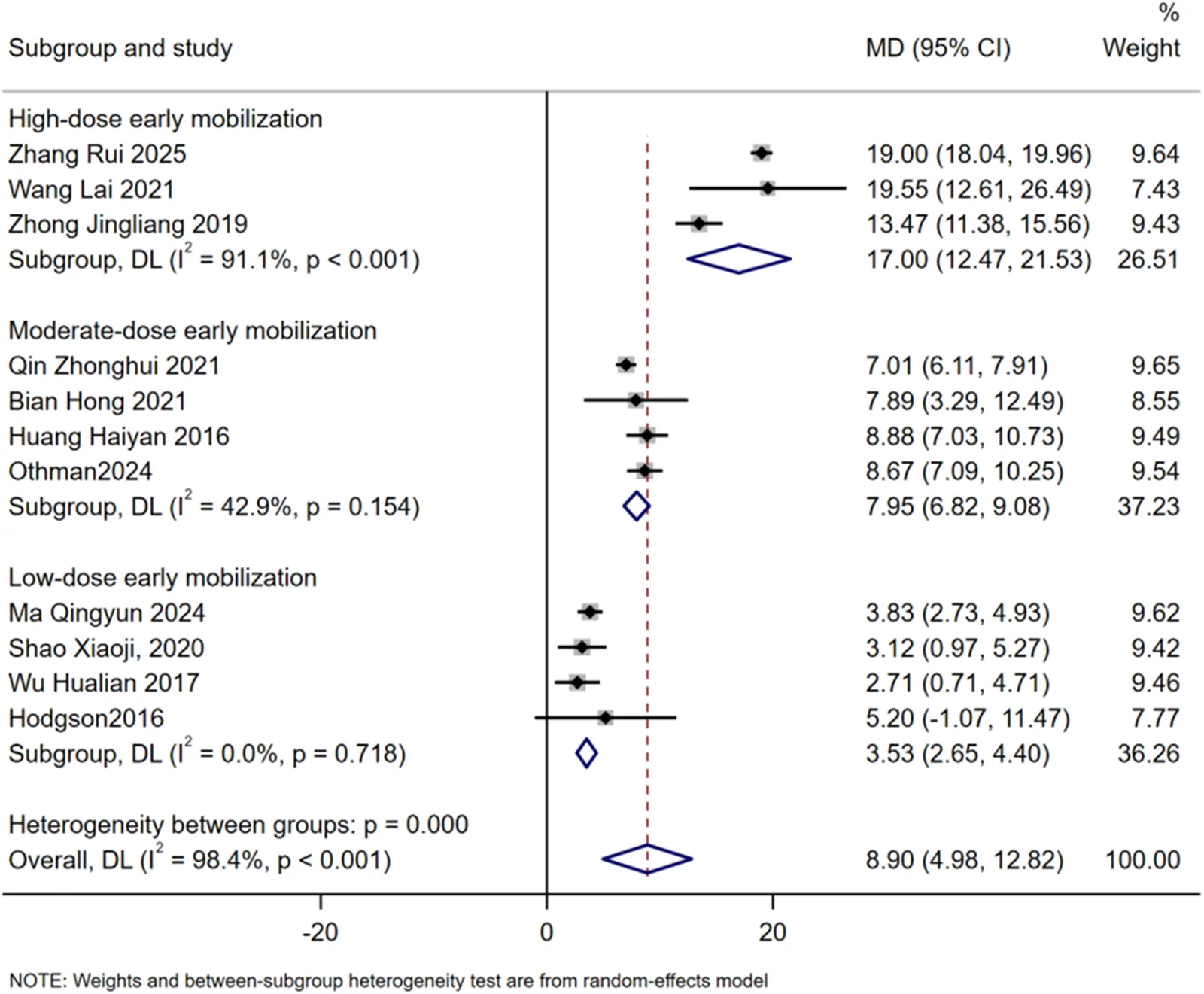
MRC forest plot.

Similarly, early mobilization significantly improved Medical Research Council (MRC) scores compared with standard care [MD = 8.90, 95% CI (4.98, 12.82), *P* < 0.001; Figure 4]. Dose-stratified subgroup analysis showed a graded effect. The largest improvement was observed in the high-dose subgroup [MD = 17.00, 95% CI (12.47, 21.53), *P* < 0.001], followed by the moderate-dose subgroup [MD = 7.95, 95% CI (6.82, 9.08), *P* < 0.001] and the low-dose subgroup [MD = 3.53, 95% CI (2.65, 4.40), *P* < 0.001].

The heterogeneity test indicated substantial overall heterogeneity (*I*^2^ = 98.4%, *P* < 0.001). Within-subgroup heterogeneity was high in the high-dose subgroup (*I*^2^ = 91.1%, *P* < 0.001), moderate in the moderate-dose subgroup (*I*^2^ = 42.9%, *P* = 0.154), and absent in the low-dose subgroup (*I*^2^ = 0.0%, *P* = 0.718). The test for between-subgroup heterogeneity was statistically significant (*P* < 0.001), suggesting that mobilization dose may partly explain the observed heterogeneity.

Regarding ICU-acquired weakness (ICU-AW), pooled analysis showed that early mobilization significantly reduced the risk of ICU-AW compared with standard care [RR = 0.35, 95% CI (0.26, 0.47), *P* < 0.001; Supplementary Figure S2]. Dose-stratified subgroup analysis showed that the reduction in ICU-AW risk varied across dose subgroups. The moderate-dose subgroup showed the greatest reduction [RR = 0.19, 95% CI (0.09, 0.41)], followed by the high-dose subgroup [RR = 0.31, 95% CI (0.19, 0.50)] and the low-dose subgroup [RR = 0.56, 95% CI (0.37, 0.85)].

The heterogeneity test indicated low to moderate overall heterogeneity (*I*^2^ = 28.9%, *P* = 0.188). No heterogeneity was observed within the high-dose subgroup (*I*^2^ = 0.0%, *P* = 0.459), moderate-dose subgroup (*I*^2^ = 0.0%, *P* = 0.528), or low-dose subgroup (I² = 0.0%, *P* = 0.599). The test for between-subgroup heterogeneity was statistically significant (*P* = 0.031), suggesting a possible dose-related difference in the effect of early mobilization on ICU-AW.

### 3.5 Effects on clinical outcomes

For the duration of mechanical ventilation, twenty-two studies were included. Random-effects meta-analysis showed that early mobilization significantly shortened the duration of mechanical ventilation compared with standard care. Dose-stratified analysis showed that all dose categories favored early mobilization, with the high-dose subgroup showing the greatest reduction.

For ICU length of stay, twenty-two studies were included. Random-effects meta-analysis showed that early mobilization was associated with a significantly shorter ICU stay. Subgroup analysis demonstrated reductions across all dose categories, with the moderate-dose subgroup showing the most favorable effect.

For total hospital length of stay, ten studies were included. Random-effects meta-analysis showed that early mobilization significantly reduced hospital length of stay compared with standard care. Dose-stratified analysis showed significant reductions across all dose categories, with the high-dose subgroup showing the greatest reduction. Detailed results are presented in Table 2 and Supplementary Figures S3–S5.

**Table 2:** Summary of meta-analysis and dose-stratified subgroup analyses for clinical outcomes.

Outcome	Dose group	Studies, n	Heterogeneity		Model	Effect estimate	
			*P*	I^2^ (%)		Effect estimate (95%CI)	*P* value
mechanical ventilation	Overall	22	<0.001	96.0	random	MD = −2.18, 95% CI (−2.62, −1.73)	<0.001
	high-dose	5	<0.001	87.7	random	MD = −2.72, 95% CI (−3.49, −1.95)	<0.001
	moderate-dose	9	<0.001	89.1	random	MD = −2.17, 95% CI (−2.65, −1.69)	<0.001
	low-dose	8	<0.001	96.0	random	MD = −1.87, 95% CI (−2.57, −1.17)	<0.001
ICU length of stay	Overall	22	<0.001	91.5	random	MD = −3.69, 95% CI (−4.24, −3.14)	<0.001
	high-dose	5	<0.001	87.8	random	MD = −3.39, 95% CI (−4.58, −2.20)	<0.001
	moderate-dose	9	<0.001	83.5	random	MD = −4.14, 95% CI (−5.00, −3.27)	<0.001
	low-dose	8	<0.001	95.7	random	MD = −3.45, 95% CI (−4.53, −2.36)	<0.001
total hospital length of stay	Overall	10	<0.001	90.0	random	MD = −4.19, 95% CI (−5.66, −2.72)	<0.001
	high-dose	4	<0.001	92.6	random	MD = −5.59, 95% CI (−9.40, −1.77)	0.004
	moderate-dose	3	0.455	0.0	random	MD = −4.66, 95% CI (−5.24, −4.08)	<0.001
	low-dose	3	0.158	45.9	random	MD = −2.49, 95% CI (−3.62, −1.36)	<0.001
Diaphragmatic Function	DTF	4	<0.001	87.5	random	MD = 6.54, 95% CI (3.04, 10.04)	<0.001
	DTee	3	<0.001	94.3	random	MD = 0.01, 95% CI (−0.01, 0.04)	0.235
	DTei	3	<0.001	94.6	random	MD = 0.03, 95% CI (0.00, 0.05)	0.036
Adverse Events	Overall	13	<0.001	62.3	random	RR = 0.41, 95% CI (0.22, 0.76)	0.001
	high-dose	3	0.047	67.3	random	RR = 0.85, 95% CI (0.21, 3.54)	0.822
	moderate-dose	6	0.513	0.0	random	RR = 0.41, 95% CI (0.25, 0.67)	<0.001
	low-dose	4	0.780	0.0	random	RR = 0.25, 95% CI (0.11, 0.57)	0.001
Mortality	Overall	4	0.258	25.7	random	RR = 1.13, 95% CI (0.89, 1.42)	0.258

### 3.6 Diaphragmatic function

Four studies evaluated diaphragm thickness fraction (DTF). Random-effects meta-analysis showed that early mobilization significantly improved DTF compared with usual care.

Three studies reported diaphragm thickness at the end of expiration (DTee). Random-effects meta-analysis showed no significant difference in DTee between the early mobilization and usual care groups.

Three studies reported diaphragm thickness at the end of inspiration (DTei). Random-effects meta-analysis showed that early mobilization was associated with a small but statistically significant increase in DTei compared with usual care. Detailed results are presented in Table 2 and Supplementary Figures S6–S8.

### 3.7 Adverse events and mortality

For adverse events, thirteen studies were included. Random-effects meta-analysis showed that early mobilization was associated with a significantly lower incidence of adverse events compared with standard care. Dose-stratified analysis showed that both moderate- and low-dose subgroups were associated with a reduced incidence of adverse events, whereas no significant difference was observed in the high-dose subgroup.

For mortality, four studies were included. Meta-analysis showed no significant difference in mortality between the early mobilization and standard care groups. Detailed results are presented in Table 2 and Supplementary Figures S9–S10.

### 3.8 Publication bias

**Figure 5:**
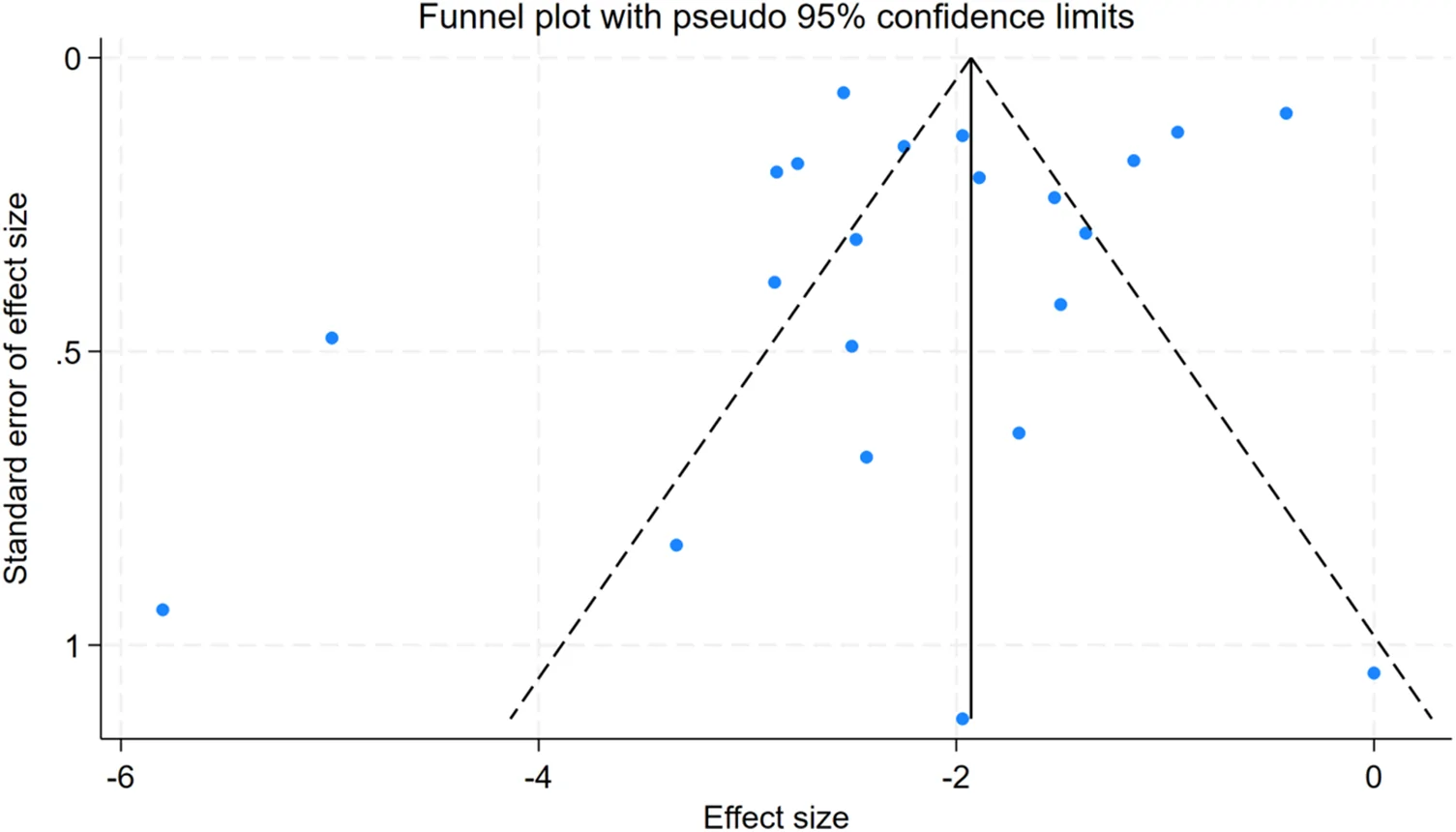
Funnel plot for publication bias assessment of mechanical ventilation duration.

The funnel plot for mechanical ventilation duration, which included the largest number of studies, is presented in Figure 5 and did not suggest substantial publication bias. Egger's and Begg's tests further confirmed the absence of significant publication bias for this outcome (all *P* > 0.05). No evidence of publication bias was observed for the remaining outcomes.

## 4 Discussion

### 4.1 Key findings

This systematic review and meta-analysis found that early mobilization improved functional status, muscle strength, diaphragmatic function, and selected clinical outcomes in mechanically ventilated adult ICU patients. Early mobilization increased Barthel Index scores, Medical Research Council scores, and diaphragmatic thickening fraction, and reduced ICU-acquired weakness, duration of mechanical ventilation, ICU length of stay, hospital length of stay, and adverse events, without significantly affecting mortality. Dose-stratified analyses showed that high-dose early mobilization had the greatest effects on Barthel Index and Medical Research Council scores, whereas moderate-dose early mobilization showed the most favorable reductions in ICU-acquired weakness and ICU length of stay. Moderate- and low-dose interventions reduced adverse events, whereas high-dose interventions did not.

Substantial heterogeneity was observed across several outcomes, indicating that dose alone cannot fully explain the variability in treatment effects. Early mobilization is a complex, multicomponent intervention rather than a single therapeutic exposure, and its effects may be influenced by intervention type, activity intensity, progression criteria, comparator intensity, patient severity, sedation practices, respiratory support, baseline functional status, and ICU context (10). Therefore, the dose-stratified findings should be interpreted as exploratory evidence supporting individualized, dose-informed mobilization rather than as a definitive prescription for an optimal dose.

### 4.2 Dose-stratified effects of early mobilization

The dose-stratified findings suggest that the association between mobilization dose and clinical outcomes may not be linear. In the present review, higher-dose mobilization was not consistently associated with greater benefits across all outcomes, and the moderate-dose subgroup showed more favorable effects on ICU-acquired weakness and ICU length of stay. This finding is clinically plausible, because critically ill patients are highly heterogeneous in terms of illness severity, baseline functional status, frailty, sedation depth, respiratory support, haemodynamic stability, and tolerance to rehabilitation (21). Therefore, it is difficult to recommend a uniform mobilization dose for all mechanically ventilated ICU patients.

Diminishing returns at higher rehabilitation doses have also been reported in previous studies. The TEAM Study Investigators reported that increased active early mobilization did not reduce hospital length of stay or mortality compared with usual ICU mobilization, but was associated with more adverse events (33). Similarly, Winkelman et al. (58) found no additional benefit of two mobilization sessions per day compared with one session, and Schaller et al. (52) reported that the achieved level and duration of mobilization, rather than frequency alone, modified outcomes in surgical critically ill patients. These findings suggest that excessive or insufficiently individualized mobilization may not provide additional benefit for all mechanically ventilated ICU patients. Instead, early mobilization should be prescribed according to patient tolerance, safety criteria, rehabilitation goals, and available clinical resources.

### 4.3 Clinical implications

The pursuit of an accurate mobilization dose for critically ill patients has remained unresolved for many years and continues to limit the reproducibility and clinical applicability of ICU rehabilitation protocols (56). González-Seguel et al. (11) highlighted important weaknesses in the reporting of mobilization dose, particularly regarding intensity, individualization, and progression. This problem was also evident in the present review. Unlike pharmacological interventions, the dose of early mobilization cannot be defined by a single parameter. It is shaped by frequency, duration, intensity, activity level, progression criteria, safety tolerance, and patient-specific factors such as illness severity, ventilatory support, sedation depth, haemodynamic stability, frailty, and baseline function (57). By classifying early mobilization into high-, moderate-, and low-dose categories, our review attempted to address this gap and found that mobilization dose may partly explain the heterogeneity in some outcomes, particularly functional outcomes and hospital length of stay.

Therefore, the FITT principle—frequency, intensity, time, and type—may provide a useful framework for prescribing and reporting mobilization dose in ICU rehabilitation (23, 59). In this review, the observed dose-response pattern likely reflected the combined effects of mobilization intensity, duration, activity level, and progression rather than frequency alone. Future trials should move beyond reporting only the highest level of mobilization achieved and should systematically document frequency, session duration, activity intensity, progression criteria, reasons for dose modification or interruption, and patient tolerance. For clinical practice, early mobilization should be dose-informed and individualized rather than uniformly high-intensity, with the goal of delivering sufficient rehabilitation stimulus while avoiding excessive treatment burden and adverse events.

In summary, the effects of early mobilization in mechanically ventilated ICU patients may be modified by mobilization dose and patient heterogeneity. This emphasizes the importance of moving beyond a uniform early mobilization strategy toward individualized, dose-informed rehabilitation protocols. Future studies should better define and report mobilization dose, including frequency, intensity, duration, activity level, progression criteria, and patient tolerance, to improve reproducibility and support more precise ICU rehabilitation practice.

### 4.4 Limitations

This study has several limitations. First, the generalizability of the findings is limited to mechanically ventilated adult ICU patients and to ICU settings similar to those represented in the included trials. Because most studies were conducted in China, the applicability of the findings to healthcare systems with different rehabilitation resources, care models, and ICU rehabilitation practices is limited.

Second, substantial heterogeneity remained across several outcomes. Differences in patient characteristics, illness severity, intervention components, comparator intensity, ICU settings, safety criteria, and definitions of early mobilization may have contributed to the observed variability. Therefore, mobilization dose alone cannot fully explain the differences in treatment effects across trials.

Third, mobilization dose was inconsistently reported across the included studies. Key dose-related elements, including frequency, intensity, duration, activity level, progression criteria, reasons for dose modification, and patient tolerance, were often incompletely described. As a result, the dose categories used in this review were based on the best available reported information and should be interpreted as an operational framework rather than externally validated dose thresholds.

Finally, blinding of participants and personnel was generally infeasible because early mobilization requires active patient participation, visible physical assistance, and direct delivery by healthcare professionals. In addition, patients are typically aware of whether they are receiving mobilization after providing informed consent and during the intervention process. This resulted in a high risk of performance bias in many trials and should be considered when interpreting the robustness of the pooled findings. Future trials should standardize the definition and reporting of mobilization dose, patient tolerance, safety events, and clinically relevant outcomes to improve comparability and reproducibility.

## 5 Conclusion

Different doses of early mobilization were associated with different patterns of benefit across outcomes in mechanically ventilated ICU patients. Moderate- to high-dose mobilization appeared to achieve more favorable effects when tailored to available clinical resources and patient tolerance. Future clinical trials should evaluate dose-adaptive mobilization protocols that dynamically adjust intervention intensity and duration according to patients' real-time physiological capacity, with the aim of improving therapeutic outcomes and optimizing the use of critical care rehabilitation resources.
