# Supplementary material for: Dose reporting and dose-stratified effects of early mobilization in mechanically ventilated adult ICU patients: a systematic review and meta-analysis
Source: Front Med (Lausanne). 2026 Aug 31;13:1920374. doi: 10.3389/fmed.2026.1920374 (PMC13572822; doi:10.3389/fmed.2026.1920374)
Supplement: Supplementary file 1 [file Table1.docx]

***Supplementary Material***

**1 Supplementary Data**

**Supplementary Table S1. Search strategies for database retrieval.**

| Database | Search strategy |
| --- | --- |
| **SinoMed: 321** | ((“重症监护病房”[不加权:扩展]) OR (“危重症监护病房”[常用字段:智能] OR “监护室”[常用字段:智能] OR “重症医学科”[常用字段:智能] OR “重症监护室”[常用字段:智能] OR “重症监护单元”[常用字段:智能] OR “加强监护病房”[常用字段:智能] OR “重症监护治疗病房”[常用字段:智能] OR “危重症”[常用字段:智能] OR “危重患者”[常用字段:智能])) AND ((“呼吸, 人工”[不加权:扩展]) OR (“机械通气”[常用字段:智能] OR “有创机械通气”[常用字段:智能] OR “人工呼吸”[常用字段:智能] OR “呼吸机”[常用字段:智能] OR “呼吸机辅助通气”[常用字段:智能] OR “机械通气患者”[常用字段:智能])) AND ((“早期活动”[常用字段:智能] OR “早期下床活动”[常用字段:智能] OR “早期康复”[常用字段:智能] OR “早期康复训练”[常用字段:智能] OR “早期功能锻炼”[常用字段:智能] OR “早期离床活动”[常用字段:智能] OR “床旁活动”[常用字段:智能] OR “渐进性活动”[常用字段:智能] OR “物理治疗”[常用字段:智能]) OR (“运动(Motion)”[不加权:扩展])) AND ((“随机对照试验”[不加权:扩展]) OR (“随机对照实验”[常用字段:智能] OR “随机对照研究”[常用字段:智能] OR “RCT”[常用字段:智能] OR “随机对照”[常用字段:智能] OR “随机”[常用字段:智能])) |
| **PubMed: 60** | ((((("Intensive Care Units"[Mesh]) OR (((Intensive Care Unit[Title/Abstract]) OR (Unit, Intensive Care[Title/Abstract])) OR (ICU Intensive Care Units[Title/Abstract]))) AND (("Respiration, Artificial"[Mesh]) OR (((((((Artificial Respiration[Title/Abstract]) OR (Artificial Respirations[Title/Abstract])) OR (Respirations, Artificial[Title/Abstract])) OR (Ventilation, Mechanical[Title/Abstract])) OR (Mechanical Ventilations[Title/Abstract])) OR (Ventilations, Mechanical[Title/Abstract])) OR (Mechanical Ventilation[Title/Abstract])))) AND (("Early Ambulation"[Mesh]) OR ((((((((Ambulation, Early[Title/Abstract]) OR (Accelerated Ambulation[Title/Abstract])) OR (Ambulation, Accelerated[Title/Abstract])) OR (Early Mobilization[Title/Abstract])) OR (Mobilization, Early[Title/Abstract])) OR (Early Mobility[Title/Abstract])) OR (Early Mobilities[Title/Abstract])) OR (Mobility, Early[Title/Abstract])))) AND (("Rehabilitation"[Mesh]) OR (Habilitation[Title/Abstract]))) AND (randomized controlled trial[Publication Type] OR randomized[Title/Abstract] OR placebo[Title/Abstract]) |
| **Embase: 50** | #14 #3 AND #6 AND #9 AND #12 AND #13 50 #13 'randomized controlled trial':ab,ti OR 'randomized':ab,ti OR 'placebo':ab,ti OR 'rct':ab,ti 1638049 #12 #10 OR #11 963403 #11 'habilitation':ab,ti 2132 #10 rehabilitation 962189 #9 #7 OR #8 15772 #8 'ambulation, early':ab,ti OR 'accelerated ambulation':ab,ti OR 'ambulation, accelerated':ab,ti OR 'early mobilization':ab,ti OR 'mobilization, early':ab,ti OR 'early mobility':ab,ti OR 'early mobilities':ab,ti OR 'mobility, early':ab,ti 8970 #7 early AND ambulation 7576 #6 #4 OR #5 130681 #5 'artificial respiration':ab,ti OR 'artificial respirations':ab,ti OR 'respirations, artificial':ab,ti OR 'ventilation, mechanical':ab,ti OR 'mechanical ventilations':ab,ti OR 'ventilations, mechanical':ab,ti OR 'mechanical ventilation':ab,ti 122425 #4 respiration, AND artificial 13544 #3 #1 OR #2 306417 #2 'intensive care unit':ab,ti OR 'unit, intensive care':ab,ti OR 'icu intensive care units':ab,ti 243936 #1 intensive AND ('care'/exp OR care) AND units 86784 |
| **Web of Science: 94** | 1: TS=(Intensive Care Units or Intensive Care Unit or Unit, Intensive Care or ICU Intensive Care Units) Results: 198265 2: TS=(Respiration, Artificial or Artificial Respiration or Artificial Respirations or Respirations, Artificial or Ventilation, Mechanical or Mechanical Ventilations or Ventilations, Mechanical or Mechanical Ventilation) Results: 79376 3: TS=(Early Ambulation or Ambulation, Early or Accelerated Ambulation or Ambulation, Accelerated or Early Mobilization or Mobilization, Early or Early Mobility or Early Mobilities or Mobility, Early) Results: 43102 4: TS=(Rehabilitation or Habilitation) Results: 307516 5: TS=(randomized controlled trial or randomized or placebo or RCT) Results: 1319302 6: #1 AND #2 AND #3 AND #4 AND #5 Results: 94 |
| **Cochrane: 47** | #1 Intensive Care Units 13370 #2 (Intensive Care Unit):ab,ti,kw OR (Unit, Intensive Care):ab,ti,kw OR (ICU Intensive Care Units):ab,ti,kw 28114 #3 #1 OR #2 32857 #4 Respiration, Artificial 6254 #5 (Artificial Respiration):ab,ti,kw OR (Artificial Respirations):ab,ti,kw OR (Respirations, Artificial):ab,ti,kw OR (Ventilation, Mechanical):ab,ti,kw OR (Mechanical Ventilations):ab,ti,kw OR (Ventilations, Mechanical):ab,ti,kw OR (Mechanical Ventilation):ab,ti,kw 20062 #6 #4 OR #5 20130 #7 Early Ambulation 2206 #8 (Ambulation, Early):ab,ti,kw OR (Accelerated Ambulation):ab,ti,kw OR (Ambulation, Accelerated):ab,ti,kw OR (Early Mobilization):ab,ti,kw OR (Mobilization, Early):ab,ti,kw OR (Early Mobility):ab,ti,kw OR (Early Mobilities):ab,ti,kw OR (Mobility, Early):ab,ti,kw 6539 #9 #7 OR #8 6680 #10 (Rehabilitation):ab,ti,kw OR (Habilitation):ab,ti,kw 84250 #11 (randomized controlled trial):ab,ti,kw OR (randomized):ab,ti,kw OR (placebo):ab,ti,kw OR (RCT):ab,ti,kw 1457427 #12 #3 AND #6 AND #9 AND #10 AND #11 47 |

**2 Supplementary Figures and Tables**

**2.1 Supplementary Figures**

Supplementary Figure S1. Risk-of-bias summary for the included randomized controlled trials.

Supplementary Figure S2. Forest plot of the effect of early mobilization on ICU-acquired weakness.

Supplementary Figure S3. Forest plot of the effect of early mobilization on duration of mechanical ventilation.

Supplementary Figure S4. Forest plot of the effect of early mobilization on ICU length of stay.

Supplementary Figure S5. Forest plot of the effect of early mobilization on total hospital length of stay.

Supplementary Figure S6. Forest plot of the effect of early mobilization on diaphragm thickness fraction.

Supplementary Figure S7. Forest plot of the effect of early mobilization on diaphragm thickness at the end of expiration.

Supplementary Figure S8. Forest plot of the effect of early mobilization on diaphragm thickness at the end of inspiration.

Supplementary Figure S9. Forest plot of the effect of early mobilization on adverse events.

Supplementary Figure S10. Forest plot of the effect of early mobilization on mortality.
